# Effects of Salt Stress on Grain Yield and Quality Parameters in Rice Cultivars with Differing Salt Tolerance

**DOI:** 10.3390/plants12183243

**Published:** 2023-09-12

**Authors:** Zhikang Li, Tianyang Zhou, Kuanyu Zhu, Weilu Wang, Weiyang Zhang, Hao Zhang, Lijun Liu, Zujian Zhang, Zhiqin Wang, Baoxiang Wang, Dayong Xu, Junfei Gu, Jianchang Yang

**Affiliations:** 1 Jiangsu Key Laboratory of Crop Genetics and Physiology/Jiangsu Key Laboratory of Crop Cultivation and Physiology, Agricultural College, Yangzhou University, Yangzhou 225009, Chinakyzhu@yzu.edu.cn (K.Z.); weiluwang868@yzu.edu.cn (W.W.); wyz@yzu.edu.cn (W.Z.); haozhang@yzu.edu.cn (H.Z.); ljliu@yzu.edu.cn (L.L.); zzj@yzu.edu.cn (Z.Z.);; 2 Jiangsu Co-Innovation Center for Modern Production Technology of Grain Crops, Yangzhou University, Yangzhou 225009, China; 3 Lianyungang Academy of Agricultural Science, Lianyungang 222000, China

**Keywords:** salt stress, rice starch, grain yield, rice quality, structure and physicochemical properties

## Abstract

Rice yield and grain quality are highly sensitive to salinity stress. Salt-tolerant/susceptible rice cultivars respond to salinity differently. To explore the variation in grain yield and quality to moderate/severe salinity stress, five rice cultivars differing in degrees of salt tolerance, including three salt-tolerant rice cultivars (Lianjian 5, Lianjian 6, and Lianjian 7) and two salt-susceptible rice cultivars (Wuyunjing 30 and Lianjing 7) were examined. Grain yield was significantly decreased under salinity stress, while the extent of yield loss was lesser in salt-tolerant rice cultivars due to the relatively higher grain filling ratio and grain weight. The milling quality continued to increase with increasing levels. There were genotypic differences in the responses of appearance quality to mild salinity. The appearance quality was first increased and then decreased with increasing levels of salinity stress in salt-tolerant rice but continued to decrease in salt-susceptible rice. Under severe salinity stress, the protein accumulation was increased and the starch content was decreased; the content of short branched-chain of amylopectin was decreased; the crystallinity and stability of the starch were increased, and the gelatinization temperature was increased. These changes resulted in the deterioration of cooking and eating quality of rice under severe salinity-stressed environments. However, salt-tolerant and salt-susceptible rice cultivars responded differently to moderate salinity stress in cooking and eating quality and in the physicochemical properties of the starch. For salt-tolerant rice cultivars, the chain length of amylopectin was decreased, the degrees of order of the starch structure were decreased, and pasting properties and thermal properties were increased significantly, whereas for salt-susceptible rice cultivars, cooking and eating quality was deteriorated under moderate salinity stress. In conclusion, the selection of salt-tolerant rice cultivars can effectively maintain the rice production at a relatively high level while simultaneously enhancing grain quality in moderate salinity-stressed environments. Our results demonstrate specific salinity responses among the rice genotypes and the planting of salt-tolerant rice under moderate soil salinity is a solution to ensure rice production in China.

## 1. Introduction

Rice (*Oryza sativa* L.) serves as the primary staple food for approximately 50% of the global population, and it contributes to around 21% of the total calorie intake worldwide by using 11% of the global cropland [1,2]. China is the largest producer and consumer of rice globally, with approximately 30% of the world’s rice production and 28% of the global rice consumption [3,4]. It is projected that rice production will need to increase by 20% by the year 2030 to ease the pressure of population growth and declining cropland [4,5]. However, following substantial advancements in grain yield during the latter half of the 20th century, rice productivity per unit area is currently approaching a plateau [6,7]. One alternative approach is to increase the planting area of rice. Globally, more than 331 million hectares of land are now affected by salinity and this is anticipated to increase by approximately 1.0–1.5 million hectares per year [8,9,10]. In China, approximately 99 million hectares of land are affected by salinity, of which ~15 million hectares can be potentially used for agricultural production, accounting for approximately 10% of China’s arable land [8,9,10]. Although rice is moderately sensitive to soil salinity, it is commonly used as a pioneer crop for improving salt-affected mudflat soil, because water in paddy fields aids in leaching out salts, thereby reducing the detrimental effects of salinity on crop growth [10,11]. Planting rice varieties that are tolerant to salinity in these regions could be a viable option for effectively utilizing such land and ensuring food security [12].

Salinization disrupts and hinders biochemical and physiological processes in plants, causing the increased accumulation of solutes and creating ionic and osmotic stresses [13,14]. This ultimately results in nutrient imbalances that limit the productivity and quality of these plants [15,16]. Rice has the ability to adapt to salt stress through various mechanisms such as regulating ionic balance, adjusting osmotic pressure, scavenging reactive oxygen species (ROS), balancing nutrient levels, and making developmental adjustments [17]. For example, a recent research study indicated that AT1, an atypical G protein γ subunit, inhibits the phosphorylation of aquaporins. These aquaporins may function as H_2_O_2_ exporters under alkaline stress, which suggests that enhancing ROS-scavenging ability can efficiently enhance the salt tolerance of rice [18]. Rice is particularly susceptible to salinity stress during its seedling and reproductive stages. In the seeding stage, salt stress can impede the growth and development of seedlings, resulting in reduced survival rates. This is primarily due to the osmotic stress caused by high salt levels, which inhibits photosynthesis and disrupts normal physiological processes. At the reproductive growth stage, salinity stress can suppress spike growth and development, resulting in an increased ratio of sterile florets and an increased incidence of panicle sterility [19,20,21].

With economic growth and improved living standards, there is a significant increase in the preference for rice varieties with superior grain quality [22,23]. Milling and processing quality, appearance quality, sensory quality, and nutritional quality are key factors in assessing grain quality. However, among these factors, cooking and eating quality are of utmost importance for producers, markets, and consumers [24]. Assessing the cooking and eating quality of rice is a challenging task due to its complex nature. One way to measure this quality is by analyzing certain physicochemical properties of the starch, such as gelatinization temperature (GT), apparent amylose content (AAC), and gel consistency (GC) [24,25]. These properties provide valuable insights into the texture and taste of rice. Currently, a limited number of studies have explored the impact of salt stress on rice quality and the underlying mechanisms involved. Moreover, the reported findings are inconsistent and conflicting. It was reported that there was a significant reduction in the amylose content, leading to a noticeable decline in the grain quality of the rice [26]. Sangwongchai et al. [27] investigated the impact of salt stress during the grain-filling stage on the starch’s physicochemical properties, and found that rice quality was improved under such conditions with increased granule diameter, decreased gelatinization peak temperature (*T*_p_), and onset temperature (*T*_o_). Furthermore, there is a limited understanding of the genotypic differences among rice genotypes in their responses to salinity stress.

In this study, we hypothesized that the response of rice quality to salt stress would differ among rice genotypes. We anticipated that certain cultivars may be able to maintain or even improve rice quality while still achieving a high yield level under salt stress conditions. Therefore, a two-year experiment with salt-tolerant rice cultivars Lianjian 5, Lianjian 6, and Lianjian 7 and salt-susceptible rice cultivars Wuyunjing 30 and Lianjing 7 were conducted at salinity levels of 0%, 0.1%, and 0.2%. We measured yields and yield components, rice quality such as milling and appearance quality, cooking and eating quality, starch fine structure, starch crystal stability, as well as starch pasting and thermal properties, with the aims to investigate genotypic differences between salt-tolerant and salt-susceptible genotypes of grain quality under salinity environments and explore the underlying mechanisms for improving gain quality under a salt-stressed environment.

## 2. Results

### 2.1. Yields and Yield Components

The grain yields and yield components were presented in Table 1. The salinity stress significantly decreased the yields and yield components for both salt-tolerant and salt-susceptible rice cultivars in two years. Compared with salt-susceptible rice cultivars, the yield reduction in salt-tolerant rice cultivars was smaller under salinity stress. For two years, compared with CK treatment, the yields of L5_T, L6_T, and L7_T decreased by 18.62%, 27.88%, and 16.88% under the salinity level of 0.1%, and decreased by 45.84%, 50.14%, and 40.80% under the salinity level of 0.2%. For W30_S and L7_S, the yields decreased by 49.32% and 37.77% under 0.1% salt stress, and decreased by 75.92% and 68.21% under T2 treatment (Table 1), when compared with CK treatment. Salinity stress significantly decreased the number of spikelets per panicle but with smaller effects in the percentage of filled grains in salt-tolerant cultivars. The decrease in yield of salt-susceptible rice cultivars was mainly due to the significant decrease in all yield components. There were small differences in 1000-grain weight between CK and salinity-stressed treatments (Table 1).

### 2.2. Milling Quality and Appearance Quality of Rice Grain

The milling quality and appearance quality of the rice grain were presented in Table 2. For milling quality, the percentage of brown rice, milled rice, and head milled rice of salt-tolerant cultivars increased significantly with the increasing levels of salt stress in two years. Different from salt-tolerant rice cultivars, the milling quality of salt-susceptible rice cultivars was increased significantly under the salinity level of 0.1%, and then decreased significantly under the salinity level of 0.2% (Table 2).

Opacity in rice grains, known as chalkiness, occurs due to the existence of air gaps between starch granules within the endosperm. Compared with the CK treatment, the chalky area, chalky kernel percentage, and chalkiness of salt-tolerant rice cultivars were firstly significantly decreased under the salinity level of 0.1%, and then significantly increased under the salinity level of 0.2%. With the increasing levels of salinity, the chalky area, chalky kernel percentage, and chalkiness of salt-susceptible rice cultivars increased significantly and the appearance quality deteriorated. The kernel length/breadth of five rice cultivars decreased significantly with the increasing levels of salinity (Table 2). The results are consistent between two experimental years.

### 2.3. Cooking and Eating Quality of Rice Grain

The cooking and eating quality of five rice cultivars with different salt tolerances was presented in Table 3. Compared with CK treatment, with the increasing levels of salinity, protein content, and amylose content, the amylose/amylopectin ratio of salt-tolerant rice cultivars was first decreased and then increased. But opposite results were observed for amylopectin content, starch content, and gel consistency. As a result, the lowest protein content, amylose content, and amylose/amylopectin ratio, as well as the highest amylopectin content, starch content, and gel consistency were observed at the salinity level of 0.1% followed by CK treatment and the salinity level of 0.2% (Table 3).

The effects of salt stress on the cooking and eating quality were different In salt-susceptible and salt-tolerant rice cultivars. With increasing levels of salt stress, the protein content, amylose content, and amylose/amylopectin ratio of salt-susceptible rice cultivars significantly increased, and amylopectin content, starch content, and gel consistency were significantly decreased (Table 3). This result indicates that the mild salt stress increased cooking and eating quality, while a high level of salt stress deteriorated cooking and eating quality in salt-tolerant rice cultivars. For salt-susceptible rice cultivars, salt stress significantly reduced the cooking and eating quality regardless of salt stress level.

### 2.4. Starch Granule Morphology and Size Distribution

**Figure 1:**
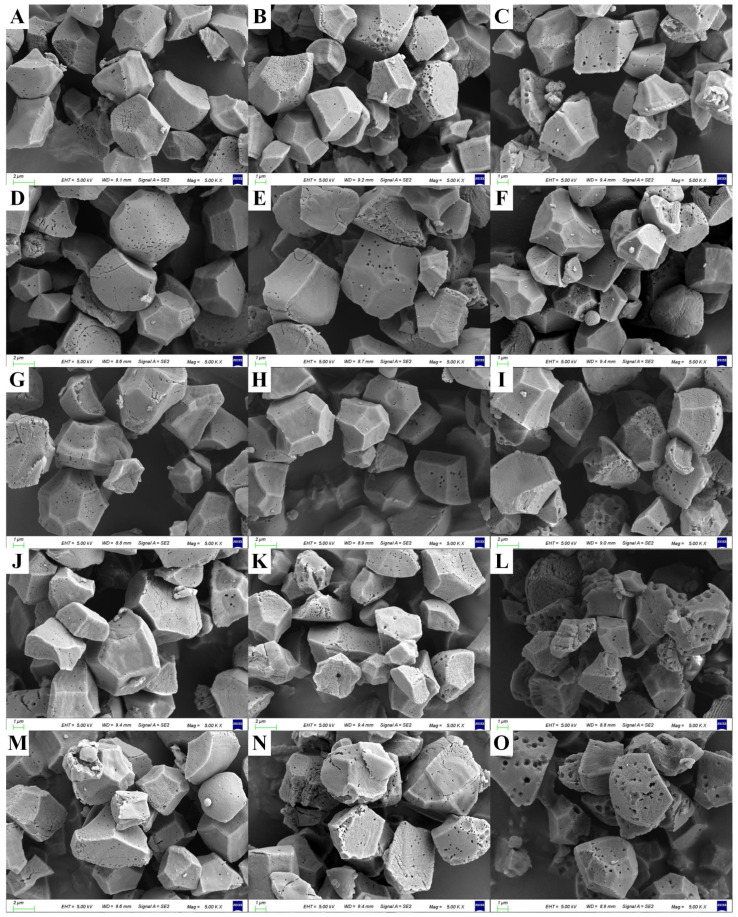
Effect of different salt stress on morphology of rice starch granules in rice cultivars with differing levels of tolerance to salt. (**A**–**C**): L5_T under CK, 0.1%_S, and 0.2%_S, respectively; (**D**–**F**): L6_T under CK, 0.1%_S, and 0.2%_S, respectively; (**G**–**I**): L7_T under CK, 0.1%_S, and 0.2%_S, respectively; (**J**–**L**): W30_S under CK, 0.1%_S, and 0.2%_S, respectively; (**M**–**O**): L7_S under CK, 0.1%_S, and 0.2%_S, respectively. EHT, extra-high tens; WD, working distance; Msg, magnifications.

In all treatments, starch granules of five cultivars displayed irregular polygonal shapes (Figure 1). In mild salt stress, the starch of salt-tolerant rice cultivars were arranged tightly in the endosperm to form larger size of starch granules, and there were smaller differences in the particle diameter of starch granules; there were fewer gaps between starch granules, when compared to the CK treatment. However, salt-tolerant rice cultivars exhibited an increased abundance of small and medium-sized starch granules under high salt stress conditions (Figure 1). For salt-susceptible rice cultivars, with increasing levels of salinity, the number of small-sized starch granules was increased with irregular and rough surfaces.

**Figure 2:**
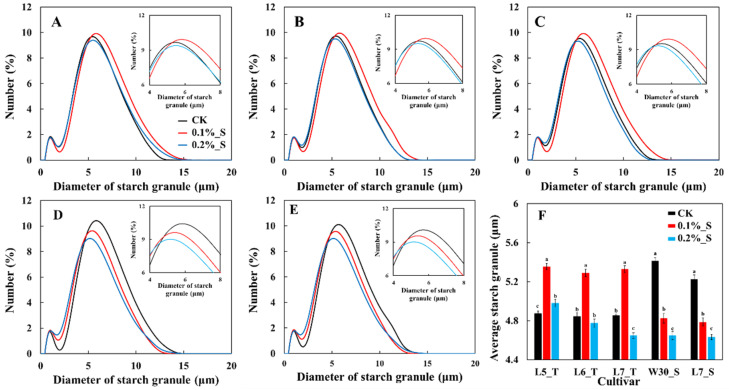
Effect of salt stress on the granule size distribution of rice starch in rice cultivars with differing levels of tolerance to salt, i.e., L5_T (**A**), L6_T (**B**), L7_T (**C**), W30_S (**D**), and L7_S (**E**), and the effects on salt stress on average size of starch granule of rice starch (**F**). Values ± SD (*n* = 3) in the same column of the same cultivar with different letters are significantly different (*p* < 0.05).

The distribution of diameter of starch granules showed a two-peak distribution with one main peak at 5–6 μm, and the distribution pattern was consistent in all treatments for all cultivars. For salt-tolerant rice cultivars, the highest peak values appeared in the salinity level of 0.1%, followed by CK and the salinity level of 0.2%, whereas for salt-susceptible rice cultivars, the highest peak values appeared in CK treatment, then followed by the salinity level of 0.1% and the salinity level of 0.2% (Figure 2). The average diameter of the starch granules was calculated (Figure 2F), and the values increased by 9.60% and decreased by 1.16% on average, when compared to the salinity level of 0.1% and the salinity level of 0.2% with CK, respectively, for salt-tolerant cultivars. Salt stress significantly decreased the average diameter of starch granules of salt-susceptible cultivars, and its value was on average decreased by 9.66% and 12.79% under the salinity level of 0.1% and the salinity level of 0.2%, respectively. These results indicate that the mild salt stress could significantly increase the volume of starch granules; however, severe salt stress decreased the size of the starch granules but not at a significant level in salt-tolerant cultivars. Salt stress significantly decreased the volume of salt-susceptible cultivars with both mild and severe salt stress.

### 2.5. Chain Length Distribution of Amylopectin

**Figure 3:**
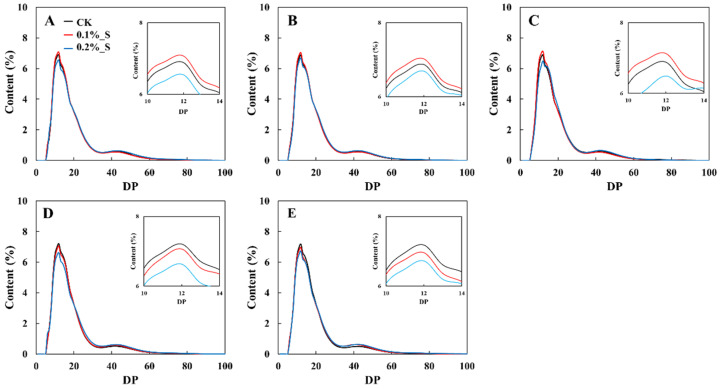
Effect of salt stress on the chain length distribution of amylopectin in rice cultivars with differing levels of tolerance to salt, i.e., L5_T (**A**), L6_T (**B**), L7_T (**C**), W30_S (**D**), and L7_S (**E**).

The chain length distribution of amylopectin is shown in Figure 3. The distribution of amylopectin chain length peaked at the degree of polymerization (DP) of 12 regardless the rice cultivars or treatments. The chain length distribution of amylopectin can be categorized into four types based on the degree of polymerization. These four types are: ‘A’ chain (DP6–12), ‘B1’ chain (DP13–25), ‘B2’ chain (DP26–37), and ‘B3’ chain (DP > 37). For salt-tolerant rice cultivars, the highest peak values appeared in the salinity level of 0.1%, followed by CK and the salinity level of 0.2%, whereas for salt-susceptible rice cultivars, the highest peak values appeared in the CK treatment, then followed by the salinity level of 0.1% and the salinity level of 0.2% (Figure 3).

For salt-tolerant cultivars, when compared with CK, the contents of the A chain, B1 chain, and degrees of branching were highest under the salinity level of 0.1%, and were lowest under the salinity level of 0.2%; while the values of the contents of the B2 chain, B3 chain, and average chain length were lowest under the salinity level of 0.1% and were highest under the salinity level of 0.2%. For salt-susceptible cultivars, with the increasing levels of salt stress, the contents of the A chain, B1 chain, and degrees of branching were continued to decrease, while the contents of the B2 chain, B3 chain, and average chain length continued to increase (Table 4).

The pattern of chain length distribution of amylopectin in response to salt stress was different in salt-tolerant and salt-susceptible cultivars. For salt-tolerant cultivars, the contents of short branch chain were increased under 0.1% salt level, but were decreased under 0.2% salt level. However, the contents of short branched-chain were decreased under salt stress regardless of the salinity levels (Table 4).

### 2.6. XRD and Structural Order of Starch under Different Levels of Salt Stress

**Figure 4:**
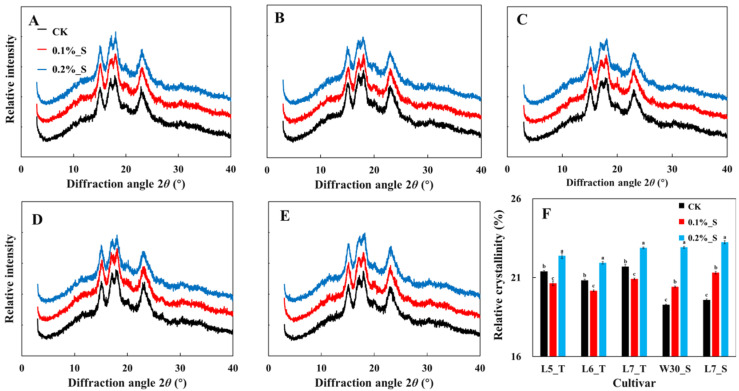
Effect of salt stress on the X-ray diffraction patterns of rice starch in rice cultivars with differing levels of tolerance to salt, i.e., L5_T (**A**), L6_T (**B**), L7_T (**C**), W30_S (**D**), and L7_S (**E**) and relative crystallinity (**F**). Values ± SD (*n* = 3) in the same column of the same cultivar with different letters are significantly different (*p* < 0.05).

There was an occurrence of an A-shaped diffraction pattern for both salt-tolerant and salt-susceptible rice cultivars under all the treatments (Figure 4). There were strong reflection peaks at 15° and 23° at a 2*θ* diffraction angle, and continuous peaks at 17° and 18°. For salt-tolerant cultivars, the crystallinity of the rice starch decreased first and then increased with the increasing levels of salt stress, while the crystallinity of salt-susceptible cultivars continued to increase with increasing levels of salinity (Figure 4).

**Figure 5:**
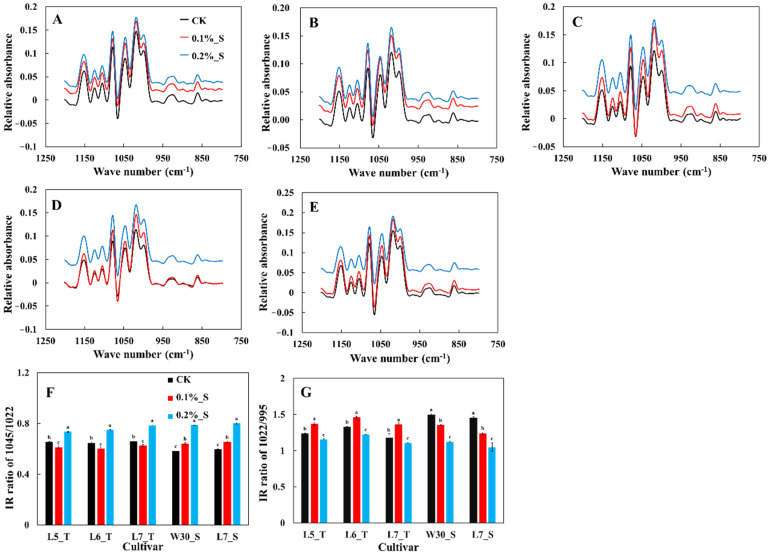
Effect of salt stress on the FTIR patterns of rice starch in rice cultivars with differing levels of tolerance to salt, i.e., L5_T (**A**), L6_T (**B**), L7_T (**C**), W30_S (**D**), and L7_S (**E**) and IR ratios of 1045/1022 (**F**) and 1022/995 (**G**). Values ± SD (*n* = 3) in the same column of the same cultivar with different letters are significantly different (*p* < 0.05).

The structural order of starch external is presented in Figure 5. The value of the band intensity ratios at 1022:995 cm^−1^ and 1045:1022 cm^−1^ reflected the content of short-range unordered molecular structure and short-range ordered molecular structure, respectively. The band intensity ratio of 1045:1022 cm^−1^ was correlated with the degree of crystallinity. With increasing levels of salt stress, the band intensity ratio of 1045:1022 cm^−1^ of salt-tolerant cultivars was decreased at the salinity level of 0.1%, and then increased significantly at the salinity level of 0.2%. For salt-susceptible cultivars, the band intensity ratio of 1045:1022 cm^−1^ continued to increase with increasing levels of salinity stress. Our result indicates that mild salt stress could reduce the crystallinity and stability of starch crystals in salt-tolerant cultivars, but increase under severe salt stress. Salt stress increased starch crystallinity and stability in both mild and severe salt stress in salt-susceptible cultivars.

### 2.7. Pasting Properties of Rice Starch under Different Salt Stress

The pasting properties of five rice cultivars under different salt stresses were shown in Table 5. Notable distinctions were observed in the pasting properties of starch between salt-tolerant and salt-susceptible cultivars. For salt-tolerant cultivars, when compared with CK, the values of peak viscosity, hot viscosity, breakdown, and final viscosity were highest under the salinity level of 0.1% and were lowest under the salinity level of 0.2%; while the values of setback, peaking time, and pasting temperature were lowest under the salinity level of 0.1% and were highest under the salinity level of 0.2%. For salt-susceptible cultivars, with the increasing levels of salt stress, the values of peak viscosity, hot viscosity, breakdown, and final viscosity of starch were decreased significantly, while the values of setback, peaking time, and pasting temperature were increased significantly.

### 2.8. Thermal Properties of Rice Starch under Different Salt Stress

The effects of salt stress on the starch thermal properties of rice were presented in Table 6. For salt-tolerant cultivars, when compared treatments of salt stress with CK treatment, the value of onset temperature (*T*_o_), peak temperature (*T*_p_), conclusion temperature (*T*_c_), ∆*H*_gel_, ∆*H*_ret,_ and R were lowest in the salinity level of 0.1%, and were highest in the salinity level of 0.2%; for salt-susceptible cultivars, those values continued to increase with the increasing levels of salinity stress.

### 2.9. Correlations between Starch Structure and Physicochemical Properties

**Figure 6:**
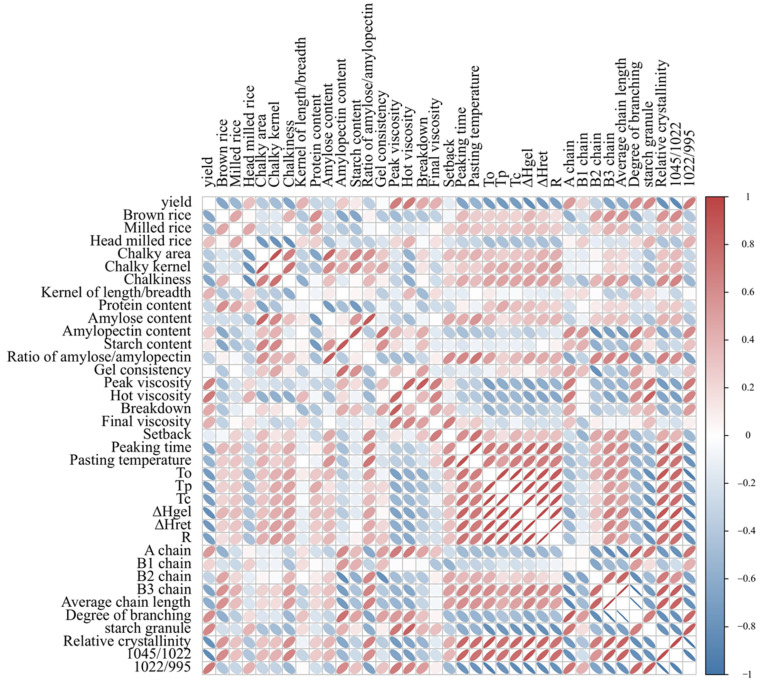
Pearson correlations for rice grain quality parameters in rice cultivars with differing levels of tolerance to salt. *T*o, onset temperature; *T*p, peak of gelatinization temperature; *T*c, conclusion temperature; Δ*H*gel, gelatinization enthalpy; Δ*H*ret, retrogradation enthalpy; R (%), retrogradation percentage (100% × Δ*H*ret/Δ*H*gel).

Figure 6 presented the correlations among various parameters of grain quality. Our findings demonstrate that relative crystallinity exhibited a negative correlation with breakdown, A and B1 chain content, peak viscosity, and final viscosity. Conversely, it showed a positive correlation with B2 and B3 chain content, gelatinization temperature, and gelatinization enthalpy. A chain content, B1 chain content, and degrees of branching were negatively correlated with the ratio of amylose/amylopectin, setback, peaking time, pasting temperature, onset temperature (*T*_o_), peak temperature (*T*_p_), conclusion temperature (*T*_c_), ∆*H*_gel_, ∆*H*_ret_, relative crystallinity and 1045/1022 cm^−1^, and were positive with peak viscosity, hot viscosity, breakdown, final viscosity, 1022/995 cm^−1^.

## 3. Discussion

### 3.1. The Effect of Salt Stress on Grain Yield and Yield Components

All yield components including the number of spikelets per panicle, the number of panicles per area, ratio of filled grains, and 1000-grain weight were significantly reduced under salt stress (Table 1), which may due to panicle sterility and the transformation of carbohydrates being limited [28,29,30,31]. The decrease in the panicle number under salt stress was the main limiting factor for yield. It is reported that the growth of rice plants was less limited under soil salt content below 1.5 g·kg^−1^ soil; most saline mudflat soils currently contain more than 3 g·kg^−1^ of salinity [12]. Rice is commonly employed as a pioneer crop for the reclamation of salt-affected mudflat soil. Irrigation helps in the leaching of salts, thereby reducing the detrimental effects of salinity on the growth and yield of the crop [10,11]. It has been estimated that there is a potential availability of over 110 million hectares of saline land worldwide. The development and utilization of these saline–alkali lands for rice cultivation would play a critical role in mitigating the challenge of insufficient cropland and ensuring food security for the projected global population, which is expected to reach 9.3 billion by the year 2050. By utilizing these previously unusable lands, we can expand agricultural production and meet the increasing demand for food in the future [32].

Yield response to salt stress varies greatly among different genotypes of rice cultivars. For salt-tolerant rice cultivars, L5_T, L6_T, and L7_T, the yields under the salinity level of 0.1% were reduced by 18.62%, 27.88%, and 16.88% on average in a two-years experiment, respectively, when compared to CK treatment. However, grain yields were significantly reduced by 49.32% and 37.77% for salt-susceptible rice cultivars W30_S and L7_S, respectively, at the salt stress level of 0.1%. Similar tends were observed at the salt level of 0.2%. The higher grain yield of salt-tolerant rice cultivars could be attributed to the differences in agronomic and physiological traits between salt-tolerant and salt-susceptible cultivars. For example, higher photosynthesis rate, higher number of productive tillers, larger root system, high Na^+^/K^+^ ratio, higher proline content, higher soluble carbohydrates content, and antioxidant enzymes activity were observed in salt-tolerant cultivars [33,34]. Some salt tolerance genes such as *OsCML15*, *OsALDH2C1*, *OsSAP16*, *OsAKT1*, *OsCLC1*, *OsNRT1*, *OsTPC1*, *OsTPKa*, etc., were also identified [33]. These advancements may help the breeders to breed salt-tolerant rice to make better use of mudflats.

### 3.2. Effect of Salt Stress on Milling and Appearance Quality for Different Salt Tolerance Rice Cultivars

The appearance qualities of rice include grain size and shape, chalkiness, transparency, of which the chalkiness is most important as it diminishes the visual appeal of rice grains and influences consumers’ preferences [35]. Under salt stress during grain-filling, the supplies of photosynthetic assimilates to grains were limited. As a result, cavities can form due to the loose arrangement of starch granules in the endosperm, and the chalkiness was attributed to the light scattering caused by these cavities [24,36]. In this study, the response of appearance quality to salt stress varied significantly among different genotypes of rice. With the increasing levels of salt stress, the chalkiness of salt-susceptible rice cultivars was increased significantly, while the chalkiness of salt-tolerant rice cultivars was decreased under mild salt stress (0.1% salt stress), and then increased under severe salt stress (0.2% salt stress) (Table 2), which is in line with findings reported in previous studies [26,27]. The variation in expression patterns of *OsSSIIa* and *OsSSIIIa*, which play a role in starch synthesis during grain filling, could be responsible for this phenomenon [37].

The milling quality of rice grain encompasses several economically significant traits, including the ratio of brown rice, milled rice, and head milled rice. Crop management practices, such as the application of organic fertilizer, irrigation, and site-specific nitrogen management, could improve milling quality under salt stress [38,39]. There were also great genetic variations in milling quality under stressed environments. Under both mild and severe salt stress conditions, salt-tolerant rice cultivars exhibited superior milling quality compared to salt-susceptible rice cultivars (Table 2). Lanning et al. [40] reported that the starch granules in the chalky area of rice grains were found to be massive or granular in structure, characterized by a porous and loose arrangement, which resulted in the reduction in grain tenacity and milling quality. Similar results were found in this study (Table 2 and Figure 1). In Figure 6, correlation analysis also showed significant negative correlation between head milled rice rate and chalky area, chalky kernel, and chalkiness (r= −0.75, −0.80, and −0.81, respectively).

### 3.3. Effect of Salt on Eating and Cooking Quality for Different Salt-Tolerant Rice Cultivars

The cooking and eating quality of rice are significantly influenced by the presence of starch (amylose and amylopectin) and protein, which make up approximately 90% of the dry weight of rice grains [36]. Under mild salt stress, amylose content, protein content, and ratio of amylose/amylopectin of salt-tolerant rice cultivars were decreased, but the amylopectin content and starch content were significantly increased. However, the opposite results were observed in salt-susceptible cultivars (Table 3). The variations in genotypes in response to salt stress could be due to the accumulation and changes in activities of starch synthetic enzymes, for example, the activity of granule-bound starch synthase and starch branching enzyme of salt-tolerant rice cultivars could be increased significantly under mild salt stress, leading to the increase in starch and amylopectin accumulation [27,41]. However, the content of starch, content of amylopectin, and gel consistency were decreased and protein content, amylose content, and ratio of amylose/amylopectin were increased in all cultivars under severe salt stress (Table 3), which suggested that the impact of severe salt stress on carbohydrate and protein metabolism and transportation is similar in both salt-susceptible and salt-tolerant rice. Generally, higher contents of amylose and protein are usually associated with a harder texture of cooked rice [42], because they can increase the heat resistance of the starch crystalline structure and restrict starch swelling and leaching during the cooking process. Consequently, these factors contribute to the development of a harder and less sticky texture in the cooked rice [43,44,45].

Recent research has indicated that the fine structure of amylopectin, along with the distribution of amylopectin chain lengths, can significantly influence the cooking and eating attributes of rice [28,36]. Amylopectin chain length distribution and fine structure were significantly related to the physicochemical properties and crystal structure of the starch, which ultimately influenced the pasting and thermal properties of starch and rice cooking and eating quality [28,46]. The distribution of chain length of amylopectin was affected by the enzyme activities and gene expression of amylopectin biosynthetic genes such as *SSI* and *SSIIa* [37,47]. For salt-tolerant rice cultivars under the salinity level of 0.1%, the contents of the A and B1 chains of amylopectin and starch granule diameter were substantially increased, whereas the contents of B2 and B3 were decreased (Table 4). An increase in the levels of short-chain molecules, a decrease in the levels of long-chain molecules can lead to the destabilization of the double helix structure. This, in turn, reduces crystallinity and promotes water absorption and cooperation within the amorphous region. As a result, the gelatinization temperature and enthalpy decrease, while viscosity and breakdown value increase [46,48]. When the percentage of short amylopectin chains (A and B1 chains) increases, it creates a higher likelihood of bonding and molecular interactions. This leads to a greater force required to separate the rice grains, resulting in a higher degree of stickiness. In contrast, the presence of longer B chains in amylopectin confers stronger intermolecular interactions, enhancing the hardness of starch granules, which ultimately contributes to the firm texture observed in cooked rice [49,50]. Under severe salt stress, rice plants increased in the content of amylopectin long chains while experiencing a decrease in the content of amylopectin short chains. The increase in the chain length distribution of amylopectin led to increased crystallinity and enhanced stability of starch crystals. Consequently, the viscosity of the rice was decreased, the pasting temperature was increased, and the overall cooking and eating quality were diminished [51,52]. The severe salt stress in our experiment deteriorated the cooking and eating quality of all rice cultivars. In conclusion, the cooking and eating quality of salt-tolerant rice were improved under mild salt stress and were deteriorated under severe salt stress. While the cooking and eating quality of salt-susceptible cultivars continued to deteriorate under salt stress (Table 5 and Table 6). By selecting salt-tolerant cultivars, it is possible to produce higher-quality rice at the expense of small yield losses in mudflat of moderate salt stress.

## 4. Materials and Methods

### 4.1. Rice Cultivation and Experimental Design

Rice plants of three salt-tolerant rice cultivars, namely Lianjian5 (L5_T), Lianjian6 (L6_T), and Lianjian7 (L7_T), as well as two salt-susceptible rice cultivars, namely Wuyunjing30 (W30_S) and Lianjing7 (L7_S), were grown at the experiment farm of Yangzhou University, Jiangsu Province, China (32°30′ N, 119°25′ E) during the rice-growing season (May–October) in the years 2019 and 2020 (Lianjian5, Lianjian6, Lianjian7 were provided by Lianyungang Academy of Agricultural Sciences, Wuyunjing30 and Lianjing7 purchased from Jiangsu Kingearth Seed CO., Ltd., Yangzhou, China). Seeds were sown on 12 May and seedlings were transplanted to pot on 12 June in both years with three hills per pot and two seedlings per hill. The soil in the experiments was sandy loam (Typic Fluvaquent, Etisol) with 24.4 g kg^−1^ organic matter, 105 mg kg^−1^ alkali-hydrolyzable N, 34.3 mg kg^−1^ Olsen-P, and 68.2 mg kg^−1^ exchangeable K. The pots were filled with 13 kg of sieved soil per pot. Before transplanting, artificial sea salt (Blue Starfish Salt Product Co., Ltd., Hangzhou, China, 94.5% NaCl, 0.11% K^+^, 0.13% Mg^2+^, 0.06% Ca^2+^ and 3.7% SO_4_^2−^) was added to each pot. For treatment of CK, moderate salt stress, and sever salt stress, 0 g, 13 g, and 26 g were added to pots, respectively, corresponding to three salt levels of 0 g·kg^−1^ (CK), 1.0 g·kg^−1^ (0.1% salt stress; 0.1%_S), and 2.0 g·kg^−1^ (0.2% salt stress; 0.2%_S). Then, 2 g urea and 0.5 g KH_2_PO_4_ (Yuntianhua CO., Ltd., Kunming, China) were added per pot before transplanting as base fertilizer. There was a total of 600 pots with forty pots as replicates for each treatment and each genotype, and the experiment was set up in the split plot design with salt treatments in main-plots, and genotypes in subplots. At 7 days after transplanting, jointing stage, and panicle differentiation stage, 1 g of urea was added to each pot to provide additional nitrogen fertilizer to support the growth and development of the rice plants. Soil salinity meter (TR-6D, Shunkeda, Beijing, China) was used to verify the soil salinity in pots to maintain the relative stability of soil salinity throughout the whole plant growth duration (Table 7). Weeds, insects, and diseases were controlled by either chemical or manual methods following local high yielding practice. Plants were harvested on 18–20 October.

### 4.2. Rice Quality

The rice processing quality, such as the brown rice percentage, milled rice percentage, head milled rice percentage, head milled rice, chalkiness degree, and chalky grain percentage, gel consistency, amylose content, and amylopectin content were evaluated following the standard methods of High Quality Paddy (GB/T 17891-1999) [53]. For all the measurements, there were more than three replicates. The protein content was determined by micro-Kjeldahl digestion, and then multiplied by 5.95.

#### 4.2.1. Starch Isolation, Granule Morphology, and Granule-Size Distribution

According to the methods of Zhou et al. [54], neutral protease (Thermo Fisher Scientific Inc, Cleveland, OH, USA) and sodium bisulfite solution (Sionpharm Chemical Reagent CO., Ltd., Shanghai, China) was used to remove protein from the rice flour first, and then the rice flour was agitated in DMSO/LiBr (Thermo Fisher Scientific Inc., Cleveland, OH, USA) to remove impurities. Ethanol (Sionpharm Chemical Reagent CO., Ltd., Shanghai, China) was added to the solution to induce the precipitation of starch. Finally, the starch was dried at 30 °C and the dried starch was then sieved through a 200-mesh sieve.

For observation of starch granule morphology, the starch was dried at 40 °C for 4 h and then coated with gold and photographed using scanning electron microscopy (GeminiSEM 300, Carl Zeiss, Oberkochen, Germany). Laser diffraction particle size analyzer (Master 2000, Malvern, England) was used to measure the starch particle distribution from 0.1 to 2000 μm.

#### 4.2.2. X-ray Diffraction (XRD) and Fourier Transform Infrared (FTIR) Analysis

The measurement and calculation of X-ray diffraction (XRD) and Fourier transform infrared (FTIR) were referred to the method of Dankar et al. [55] with some modifications. The XRD profiles of starch were obtained using a D8 advanced X-ray diffractometer (Bruker-AXS, Karlsruhe, Germany) equipped Cu-Kα filtered radiation (λ = 0.154 nm). The angle of incidence of the X-ray beam on the sample ranged from 5° to 40° (2-θ° range) at a scanning rate of 0.02 °/s. Spectra of starch samples were recorded using an FTIR spectrophotometer (670-IR + 610-IR, Varian Co. Ltd., Palo Alto, CA, USA), then gained over a 400−4000 cm^−1^ region and then the spectra were deconvolved (k factor 2.0, half width 25) to obtain the final spectrum.

#### 4.2.3. Determination of Pasting Properties and Thermal Properties

The starch rapid viscosity analyzer (RVA) profiles were carried out by rapid viscosity analyzer (Model 3D, Newport Scientific, Warriewood, Australia) and thermal properties were performed in a DSC8500 equipped with a refrigerated cooling system (Perkin Elmer, Diamond, AR, USA).

#### 4.2.4. Fluorophore-Assisted Carbohydrate Electrophoresis (FACE)

The PA-800 Plus fluorophore-assisted carbohydrate electrophoresis (FACE) system (Beckman-Coulter, Brea, CA, USA), coupled with a solid-state laser-induced fluorescence (LIF) detector and an argon–ion laser as the excitation source was used to measure the chain length distributions (CLDs) of starch. All starch samples were debranched by isoamylase (Thermo Fisher Scientific Inc., Cleveland, OH, USA) and labeled with 8-aminopyrene-1, 3, 6, trisulfonic acid (APTS, Thermo Fisher Scientific Inc., Cleveland, OH, USA) as fluorophore.

### 4.3. Statistical Analysis

The data shown in all the tables are the average of the triplicate replicates. A one-way Analysis of Variance (ANOVA) was used to determine statistically significant differences in means, and means were tested by the least significant difference at the P_0.05_ level using the SPSS 16.0 statistical software program.

## 5. Conclusions

Five rice cultivars with different salt tolerances were cultivated under three salt levels. The yields and yield components for salt-tolerant and salt-susceptible rice cultivars were decreased under salt stress, but the yield loss of salt-tolerant rice cultivars was much smaller, especially under mild salt stress. With increasing levels of salt stress, there is a notable enhancement in the milling quality for both salt-tolerant and salt-susceptible rice cultivars. Salt stress significantly reduced the appearance quality for salt-susceptible rice cultivars, but firstly improved the appearance quality in mild salt stress and then deteriorated appearance quality in severe salt stress for salt-tolerant rice cultivars. Under severe salt stress (0.2% salt stress), both salt-tolerant rice cultivars and salt-susceptible rice cultivars had increased rice protein content, long branch-chain content, and relative crystallinity, and decreased starch granule size and branch chain short branch chain content, resulting in deteriorated grain quality. However, there were genotypic differences in the responses of cooking and eating quality to mild salinity. For salt-tolerant rice cultivars, the chain length of amylopectin was decreased, the degrees of order of starch structure were decreased, and the pasting properties and thermal properties were increased significantly, whereas for salt-susceptible rice cultivars, cooking and eating quality was deteriorated under moderate salinity stress. These findings indicate that the selection of salt-tolerant rice cultivars can help maintain rice production at a relatively high level while improving grain quality in moderately saline environments.

## Figures and Tables

**Table 1:** Effects of salt stress on yield and yield components in rice cultivars with differing levels of tolerance to salt.

Year/Cultivar	Treatment	Number of Panicles per Pot	Number of Spikelets per Panicle	Total Spikelets per Pot	Percentage of Filled Grains (%)	1000-Seeds Weight (g)	Grain Yield (g pot^−1^)
2019/L5_T	CK	22.33 ± 0.58 a	167.92 ± 1.57 a	3749.66 ± 65.53 a	82.79 ± 0.23 a	23.48 ± 0.01 a	72.88 ± 1.06 a
	0.1%_S	21.67 ± 0.58 a	152.90 ± 0.77 b	3312.75 ± 81.13 b	80.23 ± 0.12 b	23.19 ± 0.04 b	61.64 ± 1.42 b
	0.2%_S	17.00 ± 1.00 b	138.98 ± 0.42 c	2362.39 ± 132.17 c	79.21 ± 0.15 c	22.65 ± 0.03 c	42.37 ± 2.27 c
2019/L6_T	CK	19.33 ± 0.58 a	155.08 ± 1.37 a	2997.67 ± 65.76 a	82.94 ± 0.21 a	27.93 ± 0.02 a	69.43 ± 1.32 a
	0.1%_S	18.33 ± 0.58 a	136.25 ± 1.54 b	2497.41 ± 51.78 b	80.34 ± 0.12 b	26.70 ± 0.02 b	53.57 ± 1.01 b
	0.2%_S	16.33 ± 0.58 b	121.48 ± 2.18 c	1983.34 ± 34.08 c	77.79 ± 0.27 c	24.20 ± 0.02 c	37.34 ± 0.48 c
2019/L7_T	CK	21.00 ± 1.00 a	125.36 ± 2.82 a	2630.81 ± 73.39 a	81.54 ± 0.48 a	27.84 ± 0.01 a	59.71 ± 1.35 a
	0.1%_S	20.00 ± 0.00 a	112.05 ± 0.63 b	2241.00 ± 12.52 b	81.02 ± 0.18 a	27.50 ± 0.02 b	49.94 ± 0.32 b
	0.2%_S	16.33 ± 0.58 b	103.80 ± 1.77 c	1694.89 ± 38.93 c	80.80 ± 0.70 a	25.95 ± 0.03 c	35.54 ± 0.71 c
2019/W30_S	CK	22.67 ± 0.58 a	154.14 ± 1.32 a	3493.26 ± 60.16 a	79.71 ± 0.20 a	25.76 ± 0.17 a	71.71 ± 0.65 a
	0.1%_S	20.67 ± 0.58 b	130.00 ± 2.27 b	2685.89 ± 32.72 b	65.94 ± 0.93 b	25.14 ± 0.01 b	44.52 ± 0.37 b
	0.2%_S	14.00 ± 0.00 c	112.00 ± 0.19 c	1568.05 ± 2.69 c	51.07 ± 0.21 c	24.48 ± 0.01 c	19.61 ± 0.10 c
2019/L7_S	CK	23.67 ± 0.58 a	154.57 ± 1.90 a	3657.51 ± 45.67 a	79.75 ± 0.21 a	25.81 ± 0.01 a	75.27 ± 0.73 a
	0.1%_S	20.67 ± 0.58 b	133.65 ± 1.28 b	2761.54 ± 51.23 b	66.08 ± 0.11 b	25.11 ± 0.01 b	45.81 ± 0.78 b
	0.2%_S	15.00 ± 0.00 c	122.00 ± 0.16 c	1830.05 ± 2.34 c	56.17 ± 0.05 c	24.90 ± 0.01 c	25.60 ± 0.02 c
2020/L5_T	CK	21.33 ± 0.58 a	166.89 ± 1.41 a	3560.28 ± 102.47 a	71.84 ± 0.16 a	24.52 ± 0.04 a	62.71 ± 1.62 a
	0.1%_S	20.67 ± 0.58 a	141.27 ± 0.89 b	2919.61 ± 88.43 b	69.54 ± 0.26 b	24.15 ± 0.01 b	49.03 ± 1.34 b
	0.2%_S	16.33 ± 0.58 b	126.01 ± 0.85 c	2058.02 ± 64.92 c	66.34 ± 0.26 c	23.05 ± 0.01 c	31.47 ± 0.87 c
2020/L6_T	CK	19.33 ± 0.58 a	179.41 ± 1.05 a	3468.21 ± 84.24 a	68.79 ± 0.15 a	26.98 ± 0.01 a	64.36 ± 1.70 a
	0.1%_S	18.33 ± 0.58 a	136.25 ± 1.54 b	2498.11 ± 89.29 b	64.48 ± 0.37 b	26.80 ± 0.01 b	43.17 ± 1.45 b
	0.2%_S	15.67 ± 0.58 b	121.48 ± 2.18 c	1904.02 ± 103.52 c	61.39 ± 0.15 c	25.30 ± 0.03 c	29.57 ± 1.54 c
2020/L7_T	CK	18.33 ± 0.58 a	158.04 ± 1.69 a	2896.85 ± 62.72 a	72.47 ± 0.10 a	27.46 ± 0.02 a	57.65 ± 1.26 a
	0.1%_S	16.67 ± 0.58 b	152.48 ± 0.16 b	2541.29 ± 88.45 b	69.49 ± 0.09 b	26.96 ± 0.02 b	47.62 ± 1.71 b
	0.2%_S	13.67 ± 0.58 c	135.12 ± 1.24 c	1846.21 ± 61.58 c	67.24 ± 0.11 c	26.41 ± 0.01 c	32.79 ± 1.08 c
2020/W30_S	CK	20.67 ± 0.58 a	186.20 ± 1.19 a	3847.61 ± 83.56 a	82.37 ± 0.23 a	27.16 ± 0.02 a	86.07 ± 1.83 a
	0.1%_S	15.67 ± 0.58 b	141.29 ± 0.06 b	2213.56 ± 56.46 b	56.74 ± 0.25 b	26.92 ± 0.01 b	33.81 ± 1.22 b
	0.2%_S	12.33 ± 0.58 c	117.54 ± 0.60 c	1449.61 ± 46.22 c	46.46 ± 0.22 c	26.60 ± 0.06 c	17.91 ± 0.76 c
2020/L7_S	CK	21.00 ± 1.00 a	171.32 ± 1.76 a	3597.50 ± 74.36 a	74.46 ± 0.09 a	27.44 ± 0.02 a	73.50 ± 3.55 a
	0.1%_S	17.67 ± 0.58 b	161.03 ± 1.25 b	2844.38 ± 60.56 b	60.56 ± 0.11 b	27.13 ± 0.04 b	46.74 ± 1.28 b
	0.2%_S	11.67 ± 0.58 c	122.00 ± 0.16 c	1423.37 ± 57.38 c	57.48 ± 0.12 c	26.57 ± 0.03 c	21.74 ± 1.07 c

Values ± SD (*n*= 3) in the same column of the same cultivar with different letters are significantly different (*p* < 0.05). Three salt-tolerant rice cultivars Lianjian5 (L5_T), Lianjian6 (L6_T), Lianjian7 (L7_T), and two salt-susceptible rice cultivars Wuyunjing30 (W30_S) and Lianjing7 (L7_S) were used in the experiment.

**Table 2:** Effects of salt stress on milling quality and appearance quality in rice cultivars with differing levels of tolerance to salt.

Year/Cultivar	Treatment	Brown Rice (%)	Milled Rice (%)	Head Milled Rice (%)	Chalky Area (%)	Chalky Kernel (%)	Chalkiness (%)	Kernel Length/ Breadth
2019/L5_T	CK	84.41 ± 0.25 c	72.62 ± 0.33 c	66.36 ± 0.12 c	15.63 ± 0.19 a	17.36 ± 0.11 a	6.56 ± 0.09 a	1.64 ± 0.01 a
	0.1%_S	85.48 ± 0.32 b	73.32 ± 0.13 b	67.25 ± 0.12 b	14.55 ± 0.26 b	16.55 ± 0.20 b	6.19 ± 0.04 b	1.62 ± 0.00 b
	0.2%_S	86.15 ± 0.13 a	74.67 ± 0.27 a	68.21 ± 0.18 a	14.73 ± 0.23 b	17.17 ± 0.35 a	6.32 ± 0.10 b	1.59 ± 0.01 c
2019/L6_T	CK	84.79 ± 0.06 c	70.50 ± 0.25 c	64.24 ± 0.13 c	22.83 ± 0.13 a	32.54 ± 0.13 a	9.70 ± 0.17 a	1.59 ± 0.01 a
	0.1%_S	85.52 ± 0.30 b	72.40 ± 0.24 b	66.19 ± 0.03 b	21.62 ± 0.14 c	30.38 ± 0.23 c	9.28 ± 0.07 b	1.55 ± 0.01 b
	0.2%_S	86.13 ± 0.04 a	73.61 ± 0.41 a	67.23 ± 0.11 a	22.29 ± 0.14 b	31.65 ± 0.30 b	9.54 ± 0.12 a	1.52 ± 0.00 c
2019/L7_T	CK	84.56 ± 0.09 c	71.72 ± 0.23 c	65.78 ± 0.07 c	14.49 ± 0.11 a	22.41 ± 0.05 a	4.79 ± 0.11 a	1.75 ± 0.01 a
	0.1%_S	85.46 ± 0.22 b	72.38 ± 0.11 b	66.76 ± 0.27 b	14.34 ± 0.19 a	19.60 ± 0.22 c	4.71 ± 0.06 a	1.73 ± 0.00 b
	0.2%_S	86.30 ± 0.13 a	73.48 ± 0.07 a	67.74 ± 0.15 a	14.32 ± 0.12 a	21.22 ± 0.22 b	4.75 ± 0.11 a	1.69 ± 0.01 c
2019/W30_S	CK	84.86 ± 0.24 b	72.54 ± 0.36 b	67.63 ± 0.23 b	12.53 ± 0.28 c	19.48 ± 0.21 c	2.87 ± 0.02 c	1.57 ± 0.01 a
	0.1%_S	85.17 ± 0.14 b	74.34 ± 0.20 a	68.51 ± 0.39 a	15.81 ± 0.27 b	22.38 ± 0.09 b	4.65 ± 0.33 b	1.55 ± 0.01 b
	0.2%_S	86.27 ± 0.09 a	73.84 ± 0.25 a	62.52 ± 0.23 c	21.60 ± 0.31 a	45.70 ± 0.39 a	17.53 ± 0.24 a	1.50 ± 0.01 c
2019/L7_S	CK	82.42 ± 0.25 c	70.24 ± 0.11 c	65.56 ± 0.41 b	22.42 ± 0.02v c	36.09 ± 0.38 c	3.81 ± 0.11 c	1.68 ± 0.01 a
	0.1%_S	83.45 ± 0.25 b	74.60 ± 0.38 a	66.57 ± 0.21 a	26.50 ± 0.21 b	40.34 ± 0.10 b	6.70 ± 0.07 b	1.64 ± 0.01 b
	0.2%_S	85.45 ± 0.21 a	71.54 ± 0.34 b	60.32 ± 0.18 c	33.40 ± 0.39 a	48.52 ± 0.40 a	15.50 ± 0.33 a	1.58 ± 0.01 c
2020/L5_T	CK	83.42 ± 0.15 c	70.35 ± 0.09 c	64.25 ± 0.08 c	16.63 ± 0.09 a	18.38 ± 0.09 a	7.03 ± 0.08 a	1.62 ± 0.01 a
	0.1%_S	84.35 ± 0.10 b	71.29 ± 0.14 b	65.53 ± 0.05 b	15.38 ± 0.06 c	17.36 ± 0.09 c	6.44 ± 0.10 c	1.58 ± 0.01 b
	0.2%_S	85.25 ± 0.10 a	72.58 ± 0.06 a	67.12 ± 0.10 a	16.03 ± 0.07 b	18.06 ± 0.14 b	6.75 ± 0.02 b	1.54 ± 0.01 c
2020/L6_T	CK	82.25 ± 0.10 c	68.33 ± 0.12 c	62.46 ± 0.13 c	23.61 ± 0.03 a	34.49 ± 0.29 a	10.35 ± 0.13 a	1.57 ± 0.01 a
	0.1%_S	83.38 ± 0.04 b	70.46 ± 0.20 b	64.31 ± 0.06 b	22.56 ± 0.09 c	31.36 ± 0.25 c	9.71 ± 0.05 c	1.52 ± 0.01 b
	0.2%_S	84.22 ± 0.06 a	71.70 ± 0.23 a	66.04 ± 0.07 a	23.29 ± 0.15 b	32.46 ± 0.11 b	9.93 ± 0.09 b	1.49 ± 0.01 c
2020/L7_T	CK	83.54 ± 0.10 c	71.80 ± 0.22 c	63.20 ± 0.09 c	16.37 ± 0.16 a	23.45 ± 0.20 a	5.66 ± 0.12 a	1.70 ± 0.01 a
	0.1%_S	84.33 ± 0.05 b	72.34 ± 0.17 b	64.57 ± 0.08 b	15.79 ± 0.18 b	20.46 ± 0.25 c	4.99 ± 0.06 c	1.67 ± 0.01 b
	0.2%_S	85.63 ± 0.08 a	73.49 ± 0.05 a	66.05 ± 0.09 a	16.23 ± 0.08 a	22.39 ± 0.24 b	5.21 ± 0.10 b	1.62 ± 0.01 c
2020/W30_S	CK	82.41 ± 0.27 c	70.31 ± 0.12 c	65.25 ± 0.10 b	15.56 ± 0.28 c	20.48 ± 0.16 c	3.26 ± 0.11 c	1.54 ± 0.01 a
	0.1%_S	83.43 ± 0.07 b	72.60 ± 0.10 a	66.45 ± 0.22 a	18.18 ± 0.18 b	25.42 ± 0.20 b	6.40 ± 0.12 b	1.51 ± 0.01 b
	0.2%_S	84.63 ± 0.18 a	71.38 ± 0.07 b	60.26 ± 0.11 c	25.62 ± 0.24 a	47.25 ± 0.09 a	19.30 ± 0.13 a	1.47 ± 0.01 c
2020/L7_S	CK	80.34 ± 0.25 c	68.45 ± 0.21 c	62.42 ± 0.05 b	25.41 ± 0.05 c	37.13 ± 0.14 c	4.22 ± 0.11 c	1.62 ± 0.01 a
	0.1%_S	81.27 ± 0.09 b	70.43 ± 0.07 a	64.36 ± 0.19 a	29.34 ± 0.11 b	41.48 ± 0.21 b	7.92 ± 0.25 b	1.59 ± 0.01 b
	0.2%_S	83.62 ± 0.31 a	69.24 ± 0.10 b	58.29 ± 0.06 c	36.63 ± 0.17 a	50.19 ± 0.12 a	18.14 ± 0.14 a	1.53 ± 0.02 c

Values ± SD (*n* = 3) in the same column of the same cultivar with different letters are significantly different (*p* < 0.05). Three salt-tolerant rice cultivars Lianjian5 (L5_T), Lianjian6 (L6_T), Lianjian7 (L7_T), and two salt-susceptible rice cultivars Wuyunjing30 (W30_S) and Lianjing7 (L7_S) were used in the experiment.

**Table 3:** Effects of salt stress on cooking and eating quality in rice cultivars with differing levels of tolerance to salt.

Year/Cultivar	Treatment	Protein Content (%)	Amylose Content (%)	Amylopectin Content (%)	Starch Content (%)	Ratio of Amylose/Amylopectin	Gel Consistency (mm)
2019/L5_T	CK	11.02 ± 0.12 b	14.87 ± 0.11 b	52.51 ± 0.14 b	67.38 ± 0.05 b	0.28 ± 0.00 b	62.30 ± 0.12 b
	0.1%_S	10.77 ± 0.07 c	14.32 ± 0.04 c	55.40 ± 0.25 a	69.72 ± 0.29 a	0.26 ± 0.00 c	63.96 ± 0.07 a
	0.2%_S	11.20 ± 0.06 a	15.52 ± 0.14 a	51.53 ± 0.20 c	67.05 ± 0.06 b	0.30 ± 0.00 a	61.59 ± 0.06 c
2019/L6_T	CK	9.80 ± 0.15 b	16.12 ± 0.23 a	55.22 ± 0.11 b	71.34 ± 0.12 b	0.29 ± 0.00 b	65.50 ± 0.33 b
	0.1%_S	9.71 ± 0.08 b	15.59 ± 0.04 b	57.02 ± 0.29 a	72.61 ± 0.28 a	0.27 ± 0.00 c	67.25 ± 0.11 a
	0.2%_S	10.30 ± 0.03 a	16.33 ± 0.06 a	54.86 ± 0.12 b	71.19 ± 0.15 b	0.30 ± 0.00 a	64.44 ± 0.36 c
2019/L7_T	CK	11.59 ± 0.06 b	15.28 ± 0.04 b	54.83 ± 0.17 b	70.11 ± 0.21 b	0.28 ± 0.00 b	61.35 ± 0.10 b
	0.1%_S	11.07 ± 0.06 c	14.80 ± 0.05 c	55.81 ± 0.10 a	70.62 ± 0.05 a	0.27 ± 0.00 c	65.62 ± 0.33 a
	0.2%_S	12.15 ± 0.18 a	15.81 ± 0.15 a	53.61 ± 0.26 c	69.42 ± 0.12 c	0.30 ± 0.00 a	60.23 ± 0.05 c
2019/W30_S	CK	11.78 ± 0.10 c	13.32 ± 0.10 c	58.73 ± 0.27 a	72.05 ± 0.37 a	0.23 ± 0.00 c	90.36 ± 0.09 a
	0.1%_S	12.16 ± 0.08 b	13.90 ± 0.07 b	56.42 ± 0.15 b	70.32 ± 0.14 b	0.25 ± 0.00 b	85.35 ± 0.26 b
	0.2%_S	12.56 ± 0.09 a	14.33 ± 0.07 a	55.09 ± 0.28 c	69.42 ± 0.32 c	0.26 ± 0.00 a	82.31 ± 0.12 c
2019/L7_S	CK	8.83 ± 0.08 c	16.48 ± 0.06 c	60.29 ± 0.15 a	76.76 ± 0.10 a	0.27 ± 0.00 c	84.58 ± 0.22 a
	0.1%_S	9.10 ± 0.04 b	17.34 ± 0.12 b	58.00 ± 0.00 b	75.34 ± 0.12 b	0.30 ± 0.00 b	81.57 ± 0.22 b
	0.2%_S	9.76 ± 0.11 a	17.91 ± 0.06 a	56.37 ± 0.07 c	74.28 ± 0.11 c	0.32 ± 0.00 a	78.02 ± 0.11 c
2020/L5_T	CK	10.97 ± 0.09 b	14.92 ± 0.06 b	52.52 ± 0.12 b	67.44 ± 0.12 b	0.28 ± 0.00 b	62.34 ± 0.08 b
	0.1%_S	10.75 ± 0.13 c	14.33 ± 0.13 c	55.48 ± 0.11 a	69.80 ± 0.23 a	0.26 ± 0.00 c	64.03 ± 0.15 a
	0.2%_S	11.24 ± 0.08 a	15.65 ± 0.07 a	51.46 ± 0.06 c	67.11 ± 0.04 c	0.30 ± 0.00 a	61.40 ± 0.12 c
2020/L6_T	CK	9.97 ± 0.03 b	16.05 ± 0.14 b	55.19 ± 0.07 b	71.24 ± 0.08 b	0.29 ± 0.00 b	65.29 ± 0.10 b
	0.1%_S	9.57 ± 0.10 c	15.54 ± 0.09 c	57.24 ± 0.02 a	72.78 ± 0.11 a	0.27 ± 0.00 c	67.30 ± 0.13 a
	0.2%_S	10.36 ± 0.04 a	16.27 ± 0.05 a	55.00 ± 0.18 b	71.26 ± 0.17 b	0.30 ± 0.00 a	64.82 ± 0.45 b
2020/L7_T	CK	11.59 ± 0.06 b	15.33 ± 0.05 b	54.87 ± 0.16 b	70.20 ± 0.12 b	0.28 ± 0.00 b	61.28 ± 0.04 b
	0.1%_S	11.07 ± 0.06 c	14.79 ± 0.19 c	55.88 ± 0.03 a	70.67 ± 0.17 a	0.26 ± 0.00 c	65.91 ± 0.26 a
	0.2%_S	12.18 ± 0.04 a	15.90 ± 0.17 a	53.52 ± 0.34 c	69.42 ± 0.26 c	0.30 ± 0.00 a	60.25 ± 0.10 c
2020/W30_S	CK	11.78 ± 0.20 c	13.31 ± 0.10 c	58.72 ± 0.18 a	72.03 ± 0.08 a	0.23 ± 0.00 c	90.42 ± 0.26 a
	0.1%_S	12.17 ± 0.05 b	13.87 ± 0.07 b	56.41 ± 0.02 b	70.28 ± 0.09 b	0.25 ± 0.00 b	85.26 ± 0.08 b
	0.2%_S	12.78 ± 0.05 a	14.33 ± 0.04 a	55.26 ± 0.13 c	69.59 ± 0.14 c	0.26 ± 0.00 a	82.45 ± 0.12 c
2020/L7_S	CK	8.84 ± 0.06 c	16.45 ± 0.09 c	60.31 ± 0.09 a	76.82 ± 0.06 a	0.27 ± 0.00 c	84.30 ± 0.11 a
	0.1%_S	9.05 ± 0.06 b	17.36 ± 0.15 b	58.09 ± 0.07 b	75.45 ± 0.22 b	0.30 ± 0.00 b	81.55 ± 0.17 b
	0.2%_S	9.75 ± 0.08 a	17.83 ± 0.05 a	56.44 ± 0.10 c	74.27 ± 0.08 c	0.32 ± 0.00 a	78.02 ± 0.11 c

Values ± SD (*n* = 3) in the same column of the same cultivar with different letters are significantly different (*p* < 0.05). Three salt-tolerant rice cultivars Lianjian5 (L5_T), Lianjian6 (L6_T), Lianjian7 (L7_T), and two salt-susceptible rice cultivars Wuyunjing30 (W30_S) and Lianjing7 (L7_S) were used in the experiment.

**Table 4:** Effects of salt stress on chain length distribution of amylopectin in rice cultivars with differing levels of tolerance to salt.

Year/Cultivar	Treatment	A Chain	B1 Chain	B2 Chain	B3 Chain	Average Chain Length	Degree of Branching
2019/L5_T	CK	30.32 ± 0.04 b	47.75 ± 0.04 b	10.26 ± 0.02 b	11.67 ± 0.05 b	20.12 ± 0.02 b	4.98 ± 0.01 b
	0.1%_S	31.70 ± 0.03 a	47.90 ± 0.02 a	9.72 ± 0.02 c	10.67 ± 0.08 c	19.56 ± 0.02 c	5.14 ± 0.04 a
	0.2%_S	29.25 ± 0.03 c	46.89 ± 0.02 c	10.40 ± 0.03 a	13.46 ± 0.08 a	20.99 ± 0.01 a	4.75 ± 0.02 c
2019/L6_T	CK	30.04 ± 0.03 b	48.54 ± 0.02 c	10.01 ± 0.01 b	11.40 ± 0.03 b	19.99 ± 0.01 b	5.02 ± 0.02 a
	0.1%_S	30.49 ± 0.02 a	48.74 ± 0.03 a	9.47 ± 0.03 c	11.30 ± 0.06 c	19.90 ± 0.01 c	5.02 ± 0.00 a
	0.2%_S	28.22 ± 0.03 c	47.61 ± 0.01 b	11.47 ± 0.01 a	12.71 ± 0.06 a	20.72 ± 0.01 a	4.84 ± 0.02 b
2019/L7_T	CK	29.85 ± 0.02 b	48.03 ± 0.03 c	10.20 ± 0.02 a	11.92 ± 0.02 b	20.22 ± 0.01 b	4.95 ± 0.01 b
	0.1%_S	32.00 ± 0.0 a	48.61 ± 0.01 b	9.34 ± 0.01 c	10.06 ± 0.02 c	19.21 ± 0.01 c	5.22 ± 0.01 a
	0.2%_S	28.46 ± 0.01 c	47.33 ± 0.01 a	11.91 ± 0.01 b	12.30 ± 0.03 a	20.41 ± 0.01 a	4.92 ± 0.01 c
2019/W30_S	CK	31.85 ± 0.01 a	49.86 ± 0.02 a	8.16 ± 0.01 c	10.14 ± 0.02 c	19.18 ± 0.01 c	5.23 ± 0.02 a
	0.1%_S	30.97 ± 0.03 b	49.35 ± 0.01 b	8.61 ± 0.01 b	11.07 ± 0.03 b	19.60 ± 0.02 b	5.12 ± 0.02 b
	0.2%_S	29.92 ± 0.02 c	47.51 ± 0.03 c	9.95 ± 0.01 a	12.61 ± 0.02 a	20.49 ± 0.01 a	4.88 ± 0.02 c
2019/L7_S	CK	31.75 ± 0.02 a	49.76 ± 0.02 a	8.45 ± 0.01 c	10.03 ± 0.05 c	19.21 ± 0.02 c	5.23 ± 0.03 a
	0.1%_S	31.08 ± 0.02 b	48.13 ± 0.02 c	9.41 ± 0.01 b	11.38 ± 0.04 b	19.75 ± 0.01 b	5.07 ± 0.03 b
	0.2%_S	29.73 ± 0.02 c	48.23 ± 0.02 b	9.56 ± 0.02 a	12.47 ± 0.03 a	20.40 ± 0.01 a	4.93 ± 0.02 c
Analysis of variance							
Cultivar C		**	**	**	**	**	**
Treatment (T)		**	**	**	**	**	**
C × T		**	**	**	**	**	**

Values ± SD (*n* = 3) in the same column of the same cultivar with different letters are significantly different (*p* < 0.05). **, significant at *p* < 0.01 level. Three salt-tolerant rice cultivars Lianjian5 (L5_T), Lianjian6 (L6_T), Lianjian7 (L7_T), and two salt-susceptible rice cultivars Wuyunjing30 (W30_S) and Lianjing7 (L7_S) were used in the experiment. Three salt-tolerant rice cultivars Lianjian5 (L5_T), Lianjian6 (L6_T), Lianjian7 (L7_T), and two salt-susceptible rice cultivars Wuyunjing30 (W30_S) and Lianjing7 (L7_S) were used in the experiment.

**Table 5:** Effects of salt stress on pasting properties in rice cultivars with differing levels of tolerance to salt.

Year/Cultivar	Treatment	Peak Viscosity (cP)	Hot Viscosity (cP)	Breakdown (cP)	Final Viscosity (cP)	Setback (cP)	Peaking Time (S)	Pasting Temperature (°C)
2019/L5_T	CK	3293.00 ± 7.00 b	2279.00 ± 8.89 b	1014.00 ± 15.00 b	3781.00 ± 13.45 b	488.00 ± 15.87 b	6.88 ± 0.02 b	76.99 ± 0.34 b
	0.1%_S	3473.67 ± 9.07 a	2433.67 ± 7.64 a	1040.00 ± 8.00 a	3872.67 ± 10.69 a	399.00 ± 12.53 c	6.57 ± 0.08 c	76.44 ± 0.18 c
	0.2%_S	3134.67 ± 10.60 c	2279.67 ± 6.03 b	855.00 ± 4.58 c	3792.33 ± 5.69 b	657.67 ± 16.26 a	7.22 ± 0.19 a	78.26 ± 0.10 a
2019/L6_T	CK	2975.00 ± 9.17 b	2079.00 ± 4.00 b	896.00 ± 5.29 b	3245.33 ± 10.97 b	270.33 ± 14.01 b	6.40 ± 0.08 b	74.42 ± 0.21 b
	0.1%_S	3139.67 ± 15.63 a	2140.33 ± 7.09 a	999.33 ± 13.32 a	3271.67 ± 3.21 a	132.00 ± 18.00 c	6.24 ± 0.10 b	73.49 ± 0.07 c
	0.2%_S	2763.00 ± 10.15 c	1934.00 ± 9.54 c	829.00 ± 7.55 c	3187.67 ± 8.50 c	424.67 ± 17.04 a	6.71 ± 0.09 a	76.44 ± 0.23 a
2019/L7_T	CK	2672.33 ± 10.41 b	2130.67 ± 4.93 b	541.67 ± 10.69 a	3319.33 ± 5.51 b	647.00 ± 13.11 b	6.65 ± 0.11 b	75.18 ± 0.13 b
	0.1%_S	2896.00 ± 9.54 a	2342.00 ± 7.94 a	554.00 ± 2.62 a	3344.33 ± 10.02 a	398.33 ± 16.77 c	6.24 ± 0.08 c	74.38 ± 0.06 c
	0.2%_S	2457.67 ± 11.15 c	1990.33 ± 6.03 c	467.33 ± 15.95 b	3190.67 ± 18.77 c	733.00 ± 18.25 a	7.11 ± 0.11 a	78.35 ± 0.08 a
2019/W30_S	CK	3437.67 ± 13.32 a	2320.00 ± 2.65 a	1117.67 ± 15.95 a	3352.33 ± 5.69 a	−85.33 ± 17.04 b	6.20 ± 0.03 c	73.72 ± 0.21 c
	0.1%_S	2996.67 ± 15.95 b	2107.33 ± 17.56 b	889.33 ± 11.59 b	3222.67 ± 16.07 b	226.00 ± 4.00 c	6.64 ± 0.11 b	74.24 ± 0.09 b
	0.2%_S	2844.00 ± 12.12 c	2024.00 ± 10.54 c	820.00 ± 10.82 c	3197.00 ± 8.00 c	353.00 ± 18.19 a	6.91 ± 0.03 a	75.79 ± 0.11 a
2019/L7_S	CK	3550.33 ± 8.62 a	2444.00 ± 11.79 a	1106.33 ± 13.62 a	3659.00 ± 10.15 a	108.67 ± 1.53 b	6.50 ± 0.04 b	74.41 ± 0.25 c
	0.1%_S	2946.00 ± 7.21 b	2033.00 ± 8.00 b	913.00 ± 2.00 b	3464.33 ± 10.07 b	518.33 ± 16.29 c	6.78 ± 0.19 b	77.80 ± 0.22 b
	0.2%_S	2777.33 ± 14.29 c	1901.33 ± 14.57 c	876.00 ± 13.45 c	3407.33 ± 5.03 c	630.00 ± 18.36 a	7.47 ± 0.17 a	80.55 ± 0.17 a
2020/L5_T	CK	3297.67 ± 12.90 b	2269.67 ± 21.57 b	1028.00 ± 17.06 a	3781.00 ± 7.55 b	483.33 ± 18.50 b	6.81 ± 0.12 a b	76.74 ± 0.21 b
	0.1%_S	3490.33 ± 5.86 a	2450.00 ± 9.54 a	1040.33 ± 9.02 a	3875.33 ± 14.47 a	385.00 ± 17.06 c	6.20 ± 0.67 b	76.29 ± 0.04 c
	0.2%_S	3146.33 ± 9.61 c	2251.00 ± 11.00 b	895.33 ± 18.58 b	3792.67 ± 7.77 b	646.33 ± 11.68 a	7.20 ± 0.12 a	78.26 ± 0.08 a
2020/L6_T	CK	2976.33 ± 9.71 b	2083.67 ± 5.13 b	892.67 ± 14.22 b	3226.00 ± 7.55 b	249.67 ± 14.57 b	6.40 ± 0.08 b	74.38 ± 0.15 b
	0.1%_S	3139.00 ± 12.29 a	2142.33 ± 5.69 a	996.67 ± 17.24 a	3277.00 ± 2.62 a	138.00 ± 13.00 c	6.23 ± 0.06 c	73.39 ± 0.20 c
	0.2%_S	2750.67 ± 9.87 c	1932.33 ± 9.61 c	818.33 ± 5.51 c	3195.33 ± 3.51 c	444.67 ± 6.66 a	6.65 ± 0.03 a	76.73 ± 0.18 a
2020/L7_T	CK	2662.00 ± 20.22 b	2131.00 ± 7.81 b	531.00 ± 15.87 a	3314.00 ± 8.54 b	652.00 ± 27.71 b	6.54 ± 0.02 b	75.16 ± 0.06 b
	0.1%_S	2895.00 ± 2.65 a	2343.00 ± 1.73 a	552.00 ± 2.65 a	3353.67 ± 11.68 a	412.67 ± 6.43 c	6.32 ± 0.10 c	74.59 ± 0.12 c
	0.2%_S	2460.67 ± 5.69 c	1988.67 ± 5.51 c	472.00 ± 10.54 b	3181.33 ± 7.02 c	720.67 ± 2.52 a	7.10 ± 0.02 a	78.39 ± 0.07 a
2020/W30_S	CK	3436.00 ± 9.85 a	2318.00 ± 6.24 a	1118.00 ± 14.11 a	3350.67 ± 7.26 a	−85.33 ± 7.51 b	6.25 ± 0.03 c	73.65 ± 0.19 c
	0.1%_S	2993.67 ± 10.50 b	2112.33 ± 15.89 b	881.33 ± 8.02 b	3227.33 ± 8.14 b	233.67 ± 12.66 c	6.63 ± 0.13 b	74.34 ± 0.10 b
	0.2%_S	2841.00 ± 7.00 c	2025.00 ± 8.00 c	816.00 ± 13.00 c	3178.00 ± 10.54 c	337.00 ± 14.93 a	6.96 ± 0.02 a	75.77 ± 0.28 a
2020/L7_S	CK	3542.00 ± 15.72 a	2446.00 ± 18.25 a	1096.00 ± 25.06 a	3661.67 ± 13.65 a	119.67 ± 2.08 b	6.51 ± 0.04 c	74.32 ± 0.10 c
	0.1%_S	2942.67 ± 10.60 b	2039.00 ± 5.00 b	903.67 ± 5.69 b	3455.00 ± 6.56 b	512.33 ± 9.24 c	6.78 ± 0.10 b	77.60 ± 0.24 b
	0.2%_S	2768.33 ± 18.18 c	1901.33 ± 12.90 c	867.00 ± 27.22 b	3415.67 ± 7.09 c	647.33 ± 24.79 a	7.55 ± 0.08 a	80.36 ± 0.25 a
Analysis of variance								
Year (Y)		NS	NS	NS	NS	NS	NS	NS
Cultivar (C)		**	**	**	**	**	**	**
Treatment (T)		**	**	**	**	**	**	**
Y × C		NS	NS	*	NS	NS	NS	NS
Y × T		NS	NS	NS	NS	NS	NS	NS
C × T		**	**	**	**	**	**	**
Y × C × T		NS	NS	NS	NS	NS	NS	NS

Values ± SD (*n* = 3) in the same column of the same cultivar with different letters are significantly different (*p* < 0.05). *, significant at *p* < 0.05 level; **, significant at *p* < 0.01 level; NS, not statistically significant. Three salt-tolerant rice cultivars Lianjian5 (L5_T), Lianjian6 (L6_T), Lianjian7 (L7_T), and two salt-susceptible rice cultivars Wuyunjing30 (W30_S) and Lianjing7 (L7_S) were used in the experiment.

**Table 6:** Effects of salt stress on thermal properties of rice starch in rice cultivars with differing levels of tolerance to salt.

Year/Cultivar	Treatment	*T*_o_ (°C)	*T*_p_ (°C)	*T*_c_ (°C)	∆*H*_gel_ (J/g)	∆*H*_ret_ (J/g)	R (%)
2019/L5_T	CK	62.65 ± 0.12 b	67.47 ± 0.15 b	76.79 ± 0.13 b	9.36 ± 0.02 b	1.98 ± 0.01 b	21.15 ± 0.09 b
	0.1%_S	61.34 ± 0.03 c	66.24 ± 0.07 c	76.17 ± 0.05 c	9.10 ± 0.07 c	1.82 ± 0.02 c	19.96 ± 0.08 c
	0.2%_S	62.82 ± 0.06 a	68.02 ± 0.12 a	77.40 ± 0.08 a	9.66 ± 0.03 a	2.12 ± 0.02 a	21.92 ± 0.18 a
2019/L6_T	CK	61.35 ± 0.11 b	66.47 ± 0.03 b	75.42 ± 0.03 b	9.22 ± 0.03 b	1.92 ± 0.02 b	20.82 ± 0.15 b
	0.1%_S	60.47 ± 0.09 c	65.44 ± 0.05 c	74.35 ± 0.10 c	9.02 ± 0.02 c	1.79 ± 0.01 c	19.81 ± 0.03 c
	0.2%_S	62.03 ± 0.07 a	67.04 ± 0.07 a	76.31 ± 0.08 a	9.47 ± 0.03 a	2.01 ± 0.02 a	21.20 ± 0.11 a
2019/L7_T	CK	63.83 ± 0.04 b	68.85 ± 0.06 b	77.64 ± 0.18 b	9.48 ± 0.02 b	2.03 ± 0.02 b	21.46 ± 0.13 b
	0.1%_S	62.27 ± 0.11 c	67.34 ± 0.08 c	76.06 ± 0.04 c	9.35 ± 0.03 c	1.92 ± 0.02 c	20.56 ± 0.17 c
	0.2%_S	64.22 ± 0.10 a	69.47 ± 0.04 a	78.27 ± 0.06 a	9.82 ± 0.08 a	2.23 ± 0.02 a	22.67 ± 0.16 a
2019/W30_S	CK	60.35 ± 0.20 c	66.23 ± 0.09 c	75.48 ± 0.16 c	9.13 ± 0.02 c	1.94 ± 0.01 c	21.22 ± 0.09 c
	0.1%_S	62.42 ± 0.05 b	69.24 ± 0.10 b	77.49 ± 0.15 b	9.52 ± 0.05 b	2.07 ± 0.02 b	21.75 ± 0.23 b
	0.2%_S	64.70 ± 0.24 a	71.22 ± 0.09 a	79.23 ± 0.10 a	9.90 ± 0.03 a	2.27 ± 0.03 a	22.93 ± 0.33 a
2019/L7_S	CK	61.37 ± 0.07 c	67.14 ± 0.01 c	76.21 ± 0.05 c	9.23 ± 0.02 c	1.94 ± 0.01 c	21.01 ± 0.12 c
	0.1%_S	63.41 ± 0.06 b	68.28 ± 0.10 b	77.58 ± 0.12 b	9.54 ± 0.02 b	2.14 ± 0.02 b	22.43 ± 0.19 b
	0.2%_S	64.99 ± 0.02 a	70.25 ± 0.12 a	79.61 ± 0.06 a	9.94 ± 0.03 a	2.34 ± 0.02 a	23.57 ± 0.19 a
2020/L5_T	CK	62.64 ± 0.08 b	67.61 ± 0.07 b	76.72 ± 0.15 b	9.36 ± 0.03 b	1.98 ± 0.01 b	21.19 ± 0.09 b
	0.1%_S	61.36 ± 0.02 c	66.34 ± 0.12 c	76.11 ± 0.12 c	9.07 ± 0.05 c	1.81 ± 0.01 c	19.99 ± 0.23 c
	0.2%_S	62.83 ± 0.03 a	68.07 ± 0.06 a	77.42 ± 0.05 a	9.71 ± 0.03 a	2.11 ± 0.01 a	21.77 ± 0.14 a
2020/L6_T	CK	61.37 ± 0.14 b	66.56 ± 0.09 b	75.34 ± 0.09 b	9.22 ± 0.01 b	1.93 ± 0.01 b	20.90 ± 0.14 b
	0.1%_S	60.49 ± 0.10 c	65.42 ± 0.06 c	74.37 ± 0.17 c	9.01 ± 0.02 c	1.78 ± 0.01 c	19.79 ± 0.03 c
	0.2%_S	62.08 ± 0.08 a	67.19 ± 0.06 a	76.15 ± 0.04 a	9.49 ± 0.01 a	2.00 ± 0.02 a	21.08 ± 0.20 a
2020/L7_T	CK	63.85 ± 0.05 b	68.77 ± 0.11 b	77.76 ± 0.28 b	9.44 ± 0.03 b	2.02 ± 0.01 b	21.39 ± 0.05 b
	0.1%_S	62.28 ± 0.10 c	67.24 ± 0.09 c	76.13 ± 0.02 c	9.35 ± 0.02 c	1.92 ± 0.02 c	20.58 ± 0.20 c
	0.2%_S	64.26 ± 0.11 a	69.50 ± 0.04 a	78.24 ± 0.11 a	9.79 ± 0.09 a	2.22 ± 0.02 a	22.64 ± 0.19 a
2020/W30_S	CK	60.32 ± 0.12 c	66.25 ± 0.10 c	75.34 ± 0.04 c	9.17 ± 0.05 c	1.94 ± 0.01 c	21.16 ± 0.14 c
	0.1%_S	62.45 ± 0.05 b	69.28 ± 0.15 b	77.48 ± 0.17 b	9.56 ± 0.02 b	2.07 ± 0.01 b	21.65 ± 0.09 b
	0.2%_S	64.64 ± 0.15 a	71.20 ± 0.14 a	79.19 ± 0.04 a	9.88 ± 0.05 a	2.27 ± 0.02 a	22.95 ± 0.22 a
2020/L7_S	CK	61.37 ± 0.07 c	67.24 ± 0.09 c	76.24 ± 0.08 c	9.24 ± 0.02 c	1.93 ± 0.02 c	20.89 ± 0.16 c
	0.1%_S	63.42 ± 0.07 b	68.55 ± 0.03 b	77.61 ± 0.11 b	9.56 ± 0.02 b	2.14 ± 0.02 b	22.38 ± 0.18 b
	0.2%_S	64.91 ± 0.02 a	70.24 ± 0.10 a	79.56 ± 0.25 a	9.97 ± 0.01 a	2.34 ± 0.02 a	23.50 ± 0.16 a
Analysis of variance							
Year (Y)		NS	**	NS	NS	NS	NS
Cultivar (C)		**	**	**	**	**	**
Treatment (T)		**	**	**	**	**	**
Y × C		NS	*	NS	NS	NS	NS
Y × T		NS	NS	NS	NS	NS	NS
C × T		**	**	**	**	**	**
Y × C × T		NS	NS	NS	NS	NS	NS

Values ± SD (*n* = 3) in the same column of the same cultivar with different letters are significantly different (*p* < 0.05). *, significant at *p* < 0.05 level; **, significant at *p* < 0.01 level; NS, not statistically significant. Three salt-tolerant rice cultivars Lianjian5 (L5_T), Lianjian6 (L6_T), Lianjian7 (L7_T), and two salt-susceptible rice cultivars Wuyunjing30 (W30_S) and Lianjing7 (L7_S) were used in the experiment.

**Table 7:** Soil electrical conductivity at different plant growth durations under three salt stresses.

Year	Growth Stage	Treatment	Soil Salinity (g kg^−1^)	Soil Electrical Conductivity (μS cm^−1^)
2019	Mid-tillering stage	CK	0	227.96 ± 4.74 c
		0.1%_S	0.1	2548.65 ± 23.49 b
		0.2%_S	0.2	4869.33 ± 35.77 a
	Panicle initiation stage	CK	0	226.75 ± 4.15 c
		0.1%_S	0.1	2538.11 ± 34.49 b
		0.2%_S	0.2	4861.36 ± 18.42 a
	Heading stage	CK	0	226.45 ± 3.99 c
		0.1%_S	0.1	2539.36 ± 23.86 b
		0.2%_S	0.2	4866.237 ± 14.79 a
2020	Mid-tillering stage	CK	0	224.65 ± 3.02 c
		0.1%_S	0.1	2541.65 ± 24.20 b
		0.2%_S	0.2	4855.65 ± 35.45 a
	Panicle initiation stage	CK	0	223.51 ± 4.12 c
		0.1%_S	0.1	2537.32 ± 13.65 b
		0.2%_S	0.2	4851.30 ± 35.33 a
	Heading stage	CK	0	223.17 ± 5.10 c
		0.1%_S	0.1	2533.11 ± 27.65 b
		0.2%_S	0.2	4850.19 ± 15.12 a

Values ± SD (*n* = 3) in the same column of the same cultivar with different letters are significantly different (*p* < 0.05).

## Data Availability

The data presented in this study are available upon request from the corresponding author.
